# Predictive value of serum creatinine and total bilirubin for long-term death in patients with ischemic heart disease: A cohort study

**DOI:** 10.1371/journal.pone.0294335

**Published:** 2023-11-16

**Authors:** Zulihuma Seyiti, Long Yang, Abudushalamu Kasimujiang, Tuohutasheng Dejite, Xue-Feng Shan, Xiao-Ming Gao

**Affiliations:** 1 College of Pediatrics, Xinjiang Medical University, Urumqi, China; 2 Pediatric Cardiothoracic Surgery, First Affiliated Hospital of Xinjiang Medical University, Urumqi, China; 3 State Key Laboratory of Pathogenesis, Prevention and Treatment of High Incidence Diseases in Central Asia, Department of Cardiology of the First Affiliated Hospital of Xinjiang Medical University, Urumqi, China; 4 Xinjiang Key Laboratory of Medical Animal Model Research, Urumqi, China; 5 Clinical Medical Research Institute of Xinjiang Medical University, Urumqi, China; The University of Mississippi Medical Center, UNITED STATES

## Abstract

**Background:**

Ischemic heart disease (IHD) has a high mortality in the population. Although serum creatinine (Cr) and serum total bilirubin (TBil) are rapid and readily available biomarkers in routine blood tests, there is a lack of literature on the prognostic value of combined Cr and TBil tests for IHD. This study aimed to evaluate a combined equation based on Cr and TBil to predict the long-term risk of death in IHD and to find indicators sensitive to the prognosis of IHD patients.

**Method:**

In this study, 2625 patients with IHD were included, and the combined value and combined equations of Cr and TBil were obtained by logistic regression analysis based on Cr and TBil collected at the time of admission. Patients were divided into four groups according to the quartiles of the combined value. COX proportional hazard regression model was used to analyze the risk factors for long-term death in IHD patients. Receiver operating characteristic (ROC) curves were used to evaluate the prognostic effect of Cr, TBil and combined value on long-term death events.

**Results:**

Logistic regression analysis was performed for long-term death events with Cr and TBil as independent variables, and the logit regression model was Logit(P) = 0.0129×TBil+0.007×Cr-0.417. Multifactorial Cox regression analysis showed that high values of the equation were independent risk factors for long-term death events (all-cause death: HR 1.457, 95% CI 1.256–1.689, P<0.001; cardiovascular death: HR 1.452, 95% CI 1.244–1.695, P<0.001). Combined Cr and TBil value are more valuable in predicting long-term death (AUC: 0.609, 95% CI 0.587–0.630, P<0.001).

**Conclusion:**

Combined Cr and TBil assay is superior to single biomarkers for predicting long-term death in patients with IHD. High values of the equation are independent predictors of long-term death and can be used to identify patients at high risk for IHD.

## Introduction

The prevalence of ischemic heart disease (IHD) in the population is growing by the day, and IHD can cause severe cardiac complications and has a worldwide trend of increasing mortality [1]. Screening for sensitive and specific biological indicators, timely and accurate identification of high-risk patients, accurate assessment of their clinical prognosis, and early intervention are important to improve the prognosis of IHD patients.

Myocardial energy demand and coronary blood flow imbalance determine IHD. Most commonly, atherosclerotic lesions result in coronary artery obstruction or stenosis, blocked coronary circulation flow, and inadequate myocardial blood supply. In addition, coronary microvascular dysfunction, inflammation, and vasospasm contribute to the multifaceted and complex pathophysiological mechanisms of IHD [2, 3]. Studies suggest that high mortality in IHD may be the result of worsening metabolic risk factors, particularly high body mass index (BMI), diabetes, hypertension, high cholesterol, and renal insufficiency [4].

Elevated serum creatinine (Cr) levels are associated with an increased risk of IHD. Studies have shown that Cr may accelerate atherosclerosis by increasing the extent and number of atherosclerotic lesions, increasing the number and modification of low-density lipoprotein particles, and vascular inflammation, which can lead to IHD [5]. Bilirubin is the end product of heme degradation and is an endogenous oxidant in the body. Heme oxygenase (HO) can maintain the dynamic balance of bilirubin content in the body by regulating the synthesis and catabolism of bilirubin [6]. It has been shown that acute myocardial ischemia can activate stress processes in the body, producing oxygen radicals and oxidants that significantly increase the activity of HO-1 and eventually lead to elevated serum total bilirubin (TBil). Increased HO-1 activity in acute myocardial infarction corresponds to increased TBil levels, with a significant positive correlation between the two [7]. In conclusion, both Cr and TBil are closely related to metabolic risk factors of IHD.

Cr and TBil are rapid and readily available biomarkers in routine blood tests, but there are no literature reports on the prognostic guidance of combined Cr and TBil tests for IHD. Therefore, in this study, for the first time, the two were combined into a simplified equation to assess whether this combined value can predict long-term death in IHD patients, to find a simple and reliable adjunct to assess and predict clinical prognosis in IHD patients for early intervention to improve prognosis.

## Methods

### Research design and study population

The study was designed in strict accordance with the Strengthening the Reporting of Observational Studies in Epidemiology (STROBE) guidelines [8]. This study complied with the Declaration of Helsinki and was approved by the Ethics Committee of the First Affiliated Hospital of Xinjiang Medical University (approval number S220722-25). As this was a retrospective cohort study, follow-up was conducted by telephone. Written informed consent was obtained from individuals for the publication of any potentially identifiable images or data included in this study.

**Fig 1:**
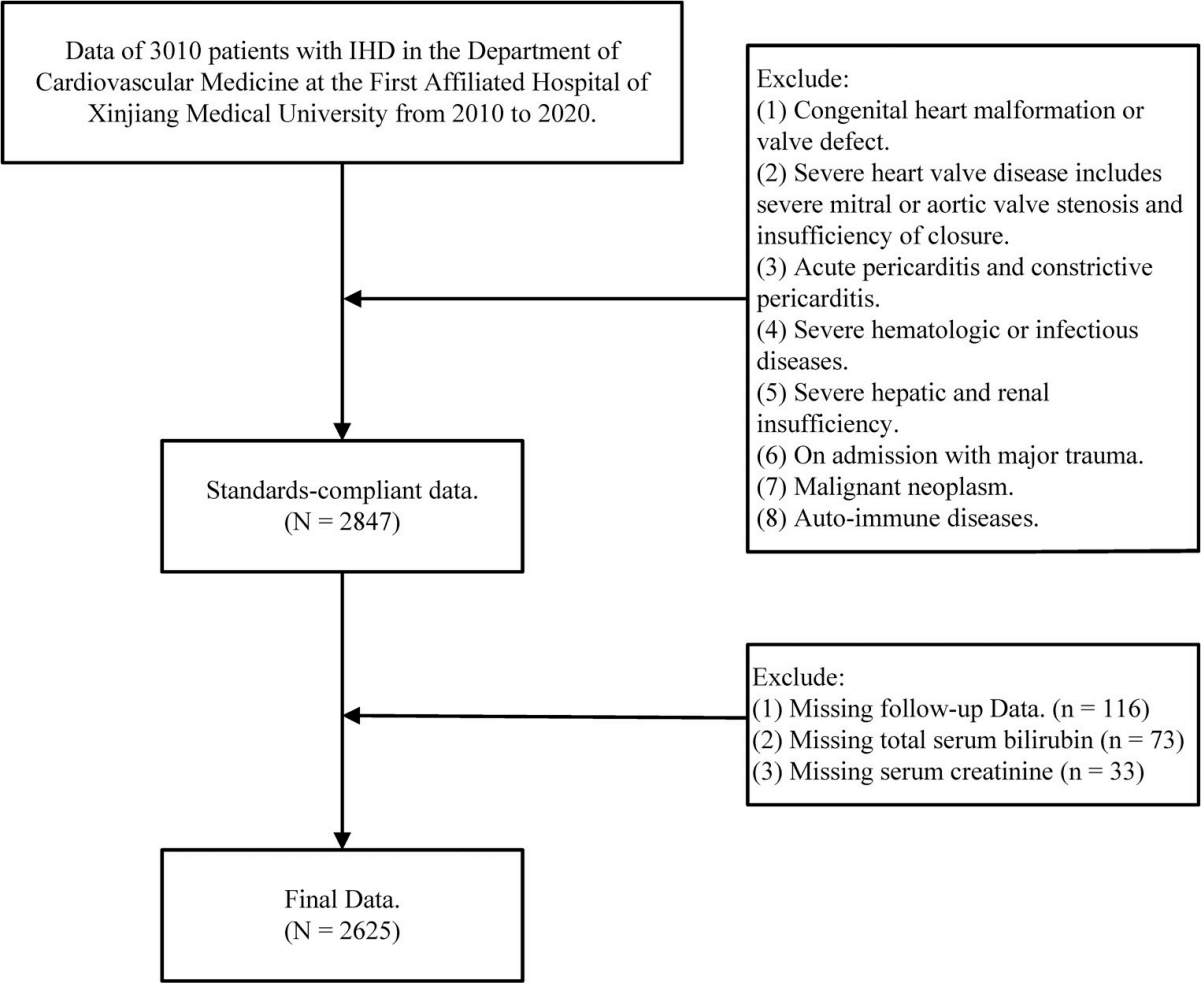
The flow chart illustrates the inclusion and exclusion criteria for patients in this study, and the entire process of data collection and follow-up.

Our research is a single-center, retrospective cohort study. The study population consisted of 3010 consecutive IHD patients admitted to the Heart Center of the First Affiliated Hospital of Xinjiang Medical University from January 2010 to December 2020. All cases met American Heart Association guidelines for the diagnosis and treatment of IHD [9]. Inclusion: Diagnosis of ischemic heart disease on admission and all of the following conditions are present: (1) Angina pectoris or equivalent symptoms during rest, manual labor, or relief with nitroglycerin. (2) The electrocardiogram on admission showed obvious signs of myocardial ischemia, and the results of the exercise test suggested the presence of myocardial ischemia. (3) Coronary angiography shows greater than 50% coronary artery stenosis. Exclusion: (1) Congenital heart malformation or valve defect. (2) Severe heart valve disease includes severe mitral or aortic valve stenosis and insufficiency of closure. (3) Acute pericarditis and constrictive pericarditis. (4) Severe hematologic or infectious diseases. (5) Severe hepatic and renal insufficiency. (6) On admission with major trauma. (7) Malignant neoplasm. (8) Auto-immune diseases. Excluding those who met the exclusion criteria, lack of clinical information and missing visits, a total of 2625 people were included in this study (**Fig 1**).

### Data collection

All patients had a detailed medical history documented by a professionally trained investigator who collected general information about the patients through electronic and paper medical records, including age, sex, body mass index (BMI), systolic and diastolic blood pressure at admission, history of smoking, history of alcohol consumption, history of hypertension, history of diabetes, history of cardiovascular disease, history of percutaneous coronary intervention (PCI), admission status (myocardial infarction, heart failure, arrhythmia), laboratory tests, and echocardiography.

### Laboratory indices and cardiogram tests

Peripheral venous blood samples were collected from all patients early in the morning on the second day of admission on an empty stomach, and complete routine blood and biochemical tests were performed. The fully automated hematology analyzer XE-5000 (Sysmex, Japan) was applied to determine hemoglobin, white blood cell count, neutrophil count and platelet count. Blood Cr, fasting blood glucose and serum triacylglycerol, TBil, total cholesterol, high-density lipoprotein cholesterol (HDL-C), low-density lipoprotein cholesterol (LDL-C) and albumin levels were measured with a fully automated biochemical analyzer VITROS 5600 (Johnson & Johnson, USA). All patients underwent echocardiography within 24 h of admission by a specialized sonographer applying an EPIQ 7C (PHILIPS, The Netherlands) color Doppler ultrasound diagnostic instrument to measure left ventricular end-diastolic diameter (LVEDD) and left ventricular ejection fraction (LVEF).

### Definition of clinical outcomes and follow-up process

The primary outcome of this study was death from any cause, and the secondary outcome was death of cardiovascular origin. Cardiovascular deaths were recorded as deaths related to myocardial infarction, congestive heart failure, sudden cardiac death, or arrhythmias. Follow-up information was collected by trained follow-up personnel who contacted the patient or the patient’s family through telephone follow-up. Death cases were obtained by confirming death certificates with patients’ families. All data were matched to hospital records. All patients were followed up by telephone from September 2022 to October 2022, of whom 2625 (87.2%) provided verbal consent and completed telephone follow-up.

### Statistical analysis

Statistical analyses of the data in this study were performed using R software (The R Foundation; http://www.r-project.org; version 4.2.1). First, binary logistic regression analysis was calculated for Cr and TBil in the sample to obtain the combined value of Cr and TBil and the combined regression equation. Patients were divided into four groups according to quartiles of combined value and baseline characteristics were compared between groups. Normality tests for all variables in this study yielded that all variables were non-normally distributed, with continuous variables expressed as medians (interquartile spacing) and categorical variables expressed as frequencies (percentages). The Mann-Whitney U test was used for all continuous variables and the chi-square test was used for all categorical variables. Kaplan-Meier survival curves were established to assess the survival rates of different groups, and the log-rank test was used to compare whether there was a significant difference in survival rates between these groups. The hazard ratios (HRs) and 95% confidence intervals (CIs) between Cr, TBil, combined value, and the incidence of all-cause death and cardiovascular death were calculated separately using Cox proportional hazards regression models. In the calculation of HRs for combined value, group 1 (Combine ≤0.236) was used as a reference. For these models, we used both unadjusted and adjusted models. Firstly, model 1 was unadjusted for any confounding factors. Second, in model 2 we adjusted for gender, age, BMI, smoking, drinking, hypertension, and diabetes. we further adjusted for albumin, LVEF, fasting plasma glucose, triglyceride, total cholesterol, and the covariates of model 2 (model 3). Finally, we also adjusted for admission of myocardial infarction, heart failure, arrhythmia, past PCI, and the covariates of model 3 (model 4). Moreover, a restricted cubic spline (RCS) analysis was performed to reflect the dose–response relationship between the combined value and the risk of the two primary outcomes. Finally, the area under the curve (AUC), sensitivity, specificity and best cut-off values of Cr, TBil and combined value were obtained by receiver operating characteristic (ROC) curve analysis to assess the predictive efficacy of the three on long-term death events. In all analyses, a two-sided P < 0.05 was considered statistically significant.

## Results

### Comparison of baseline characteristics

Among the cases between January 2010 and December 2020, we selected 3010 patients with IHD. Of these patients, 385 were excluded according to exclusion criteria. A total of 2625 patients with IHD (1974 men and 651 women) with a mean age of 65 years were finally included in this study. 1611 patients experienced all-cause death, of which 1472 were cardiovascular deaths. A flow chart of the study design is depicted in Fig 1. We derived the combined value for Cr and TBil by binary logistic regression analysis using all-cause death as the dependent variable and determined the combined equation for the combined value **(Table 1)**. We compared baseline characteristics between groups based on the quartiles of binding values, with value ≤0.236 as group 1, 0.236 < value ≤0.381 as group 2, 0.381 < value ≤0.592 as group 3, and value >0.592 as group 4 **(Table 2)**.

**Table 1:** The combined equation of logistic regression of all-cause death.

Variable	coefficient	coefficient’s SE	Z-statistic	P-value
TBil	0.013	0.004	3.452	<0.001
Cr	0.007	0.001	7.090	<0.001
Constant term	-0.417	0.114	-3.661	<0.001

Abbreviations: TBil, total bilirubin; Cr, creatinine; SE, standard error.

The formula of combined value = 0.0128637×TBil+0.0068894×Cr-0.4173822.

**Table 2:** Comparison of baseline characteristics.

Variable	Total (n = 2625)	Group1 (n = 658)	Group2 (n = 656)	Group3 (n = 654)	Group4 (n = 657)	P-value
General conditions						
Age (years)	66.00[57.00,74.00]	63.00[54.00,72.00]	65.00[56.00,73.00]	67.00[59.00,75.00]	68.00[59.00,76.00]	<0.001
Male, n (%)	1974(75.20)	402(61.09)	511(77.90)	528(80.73)	533(81.13)	<0.001
BMI (kg/m^2^)	25.33[22.86,28.08]	25.21[22.78,28.44]	25.71[23.15,28.08]	25.61[23.44,28.41]	24.74[22.32,27.38]	<0.001
SBP (mmHg)	122.00[110.00,138.00]	122.00[111.00,137.00]	121.00[110.00,135.00]	121.00[110.00,138.00]	121.00[109.00,140.00]	0.698
DBP (mmHg)	74.00[66.00,81.00]	73.00[65.00,80.00]	74.00[66.00,81.00]	74.00[66.00,82.00]	74.00[66.00,81.00]	0.693
Medical history, n (%)						
Smoking	1120(42.67)	255(38.75)	295(44.97)	287(43.88)	283(43.07)	0.113
Drinking	731(27.85)	172(26.14)	194(29.57)	179(27.37)	186(28.31)	0.558
Hypertension	1608(61.26)	381(57.90)	372(56.71)	399(61.01)	456(69.41)	<0.001
Diabetes	1452(55.31)	331(50.30)	344(52.44)	340(51.99)	437(66.51)	<0.001
Past PCI	549(20.91)	144(21.88)	146(22.26)	140(21.41)	119(18.11)	0.231
Admission conditions, n (%)						
MI	860(32.76)	210(31.91)	230(35.06)	209(31.96)	211(32.12)	0.551
HF	785(29.90)	183(27.81)	198(30.18)	205(31.35)	199(30.29)	0.553
Arrhythmia	1054(40.15)	221(33.59)	234(35.67)	299(45.72)	300(45.66)	<0.001
Laboratory test						
WBC (10^9/L)	7.05[5.77,8.51]	6.85[5.57,8.29]	7.03[5.86,8.31]	7.13[5.82,8.59]	7.15[5.76,9.09]	0.005
Plt (10^9/L)	203.00[164.00,250.00]	225.00[187.00,272.00]	204.00[169.00,243.00]	194.00[163.00,249.00]	185.00[148.00,235.00]	<0.001
Cr (umol/L)	85.00[70.00,108.00]	63.00[55.00,69.00]	79.73[73.00,86.22]	97.00[86.00,106.08]	136.00[114.00,177.52]	<0.001
UA (umol/L)	374.20[303.20,470.45]	309.20[257.00,370.00]	349.60[293.00,422.00]	389.00[322.71,473.00]	486.00[404.00,596.73]	<0.001
FPG (mmol/L)	6.39[5.02,9.32]	5.84[4.88,8.43]	6.24[4.95,8.84]	6.52[5.03,9.65]	7.11[5.40,10.18]	<0.001
TG (mmol/L)	1.22[0.89,1.73]	1.27[0.94,1.82]	1.22[0.90,1.76]	1.17[0.84,1.70]	1.19[0.88,1.65]	0.002
TC (mmol/L)	3.38[2.81,4.12]	3.51[2.92,4.34]	3.49[2.84,4.24]	3.35[2.79,4.00]	3.25[2.69,4.02]	<0.001
HDL-C (mmol/L)	0.91[0.74,1.09]	0.92[0.77,1.11]	0.94[0.78,1.11]	0.91[0.74,1.10]	0.85[0.67,1.06]	<0.001
LDL-C (mmol/L)	2.16[1.66,2.71]	2.22[1.73,2.83]	2.18[1.66,2.77]	2.12[1.65,2.67]	2.11[1.63,2.61]	0.109
TBil (umol/L)	13.99[9.80,19.90]	10.00[7.50,12.70]	13.69[10.10,17.40]	16.90[12.60,22.19]	20.10[11.60,34.33]	<0.001
Albumin (g/L)	37.70[33.90,40.70]	38.30[34.98,41.33]	38.68[34.80,41.30]	37.50[33.82,40.60]	36.10[32.27,39.32]	<0.001
LDH (U/L)	195.07[163.00,238.00]	180.00[155.00,218.00]	187.00[159.00,220.00]	201.00[166.00,251.90]	217.90[181.80,271.31]	<0.001
CK (IU/L)	69.00[46.93,103.78]	59.00[42.00,89.00]	66.60[46.60,97.00]	70.21[48.00,104.66]	85.00[52.48,131.00]	<0.001
Echocardiography						
LVEDD (mm)	61.00[56.00,66.00]	60.00[54.00,64.00]	61.00[56.00,66.00]	62.00[57.00,67.00]	62.00[57.00,67.00]	<0.001
LVEF (%)	42.00[37.00,47.35]	43.00[38.00,52.00]	42.57[37.00,47.61]	41.85[36.00,47.00]	40.00[35.00,46.00]	<0.001
Outcomes, n (%)						
All-cause death	1611(61.37)	336(51.06)	388(59.15)	391(59.79)	496(75.49)	<0.001
Cardiovascular death	1472(59.21)	303(48.48)	355(56.98)	354(57.37)	460(74.07)	<0.001
Combine	0.38[0.24,0.59]	0.16[0.10,0.20]	0.31[0.27,0.35]	0.47[0.42,0.52]	0.83[0.68,1.14]	<0.001

Abbreviations: BMI, body mass index; SBP, systolic blood pressure; DBP, diastolic blood pressure; PCI, percutaneous coronary intervention; MI, myocardial infarction; HF, heart failure; WBC, white blood cell count; Plt, platelet count; Cr, creatinine; UA, uric acid; FPG, fasting plasma glucose; TG, triglyceride; TC, total cholesterol; HDL-C, high-density lipoprotein cholesterol; LDL-C, low-density lipoprotein cholesterol; TBil, total bilirubin; LDH, lactic dehydrogenase; CK, creatine kinase; LVEDD, left ventricular end-diastolic dimension; LVEF, left ventricular ejection fraction.

Patients were divided into four groups according to the quartiles of combined value: Group 1: Combine ≤ 0.236; Group 2: 0.236 < Combine ≤ 0.381; Group 3: 0.381< Combine ≤ 0.592; Group 4: Combine > 0.592.

As shown in **Table 2**, group 4 had the oldest IHD patients among the four groups (63.00[54.00,72.00] vs. 65.00[56.00,73.00] vs. 67.00[59.00,75.00] vs. 68.00[59.00,76.00], P < 0.001), and their median left ventricular end-diastolic internal diameter (interquartile spacing) was the same as in group 3 and greater than in the remaining two groups (60.00[54.00,64.00] vs 61.00[56.00,66.00] vs 62.00[57.00,67.00] vs 62.00[57.00,67.00], P < 0.001). BMI (25.21[22.78,28.44] vs. 25.71[23.15,28.08] vs. 25.61[23.44,28.41] vs. 24.74[22.32,27.38]) and LVEF (43.00[38.00,52.00] vs. 42.57[37.00. 47.61] vs 41.85[36.00,47.00] vs. 40.00[35.00,46.00]) were the lowest. Laboratory data showed Cr levels in the four groups were 63.00[55.00,69.00], 79.73[73.00,86.22], 97.00[86.00,106.08], 136.00[114.00,177.52], TBil levels were 10.00[7.50,12.70], 13.69[10.10. 17.40], 16.90[12.60,22.19], 20.10[11.60,34.33], and the combined values were 0.16[0.10,0.20], 0.31[0.27,0.35], 0.47[0.42,0.52], and 0.83[0.68,1.14], respectively, all with statistically significant (P < 0.001). The white blood cell count, uric acid, fasting glucose, lactate dehydrogenase, and creatine kinase levels were significantly higher in group 4 compared to the other groups and showed a gradual increase from group 1 to group 4 (P < 0.01), and their platelet count, total cholesterol, HDL cholesterol, and albumin levels were lower compared to the other groups (P < 0.001), and their triglyceride levels were similar to group 3 and lower than the other two groups (P = 0.002). Group 4 had significantly higher rates of men, hypertension, diabetes mellitus, all-cause death, and cardiovascular death compared to the other groups, while arrhythmia rates were similar to group 3 and higher than the other two groups (P<0.001).

There were no statistical differences between the four groups in systolic blood pressure, diastolic blood pressure, history of smoking, history of alcohol consumption, history of PCI, admission conditions (myocardial infarction and heart failure), and LDL cholesterol.

### Kaplan-Meier survival analysis of long-term death in different combine value groups with IHD

**Fig 2:**
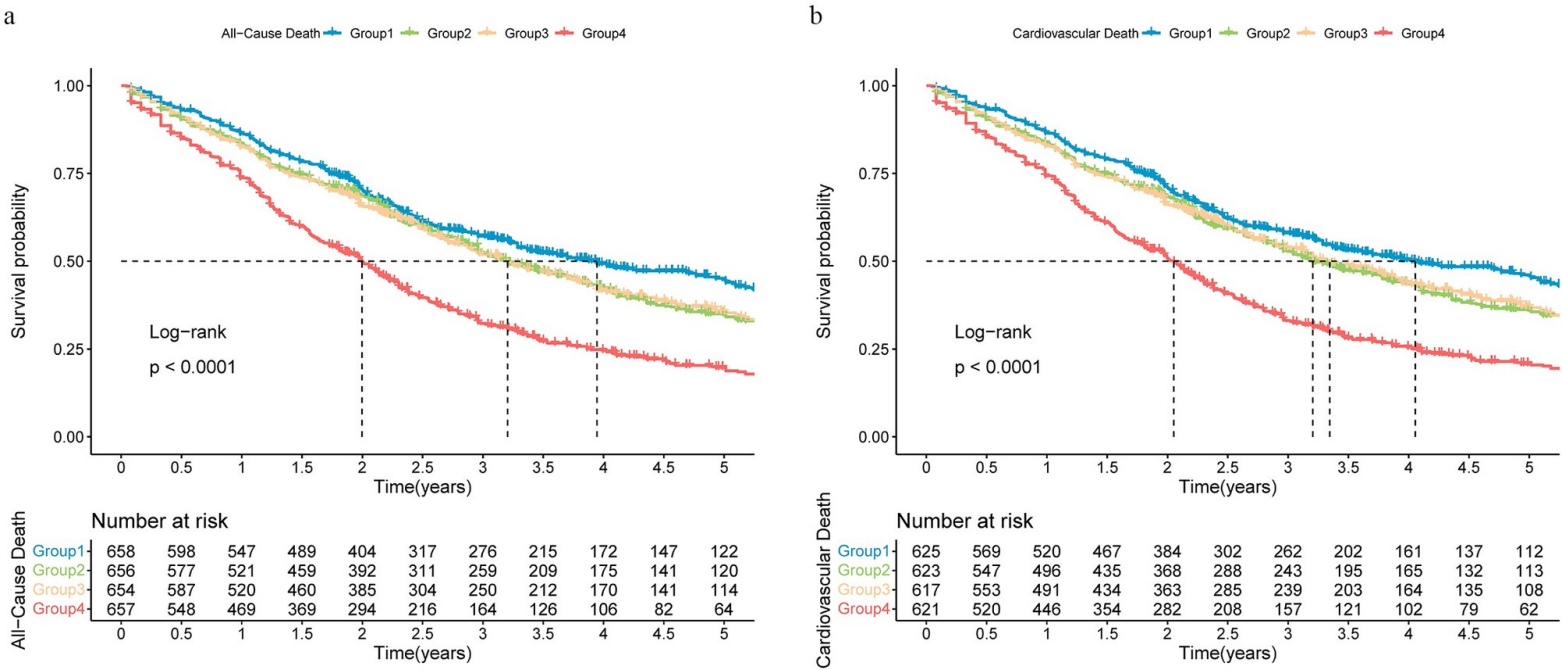
Kaplan-Meier survival rates for long-term death in different groups of IHD patients. (A) All-cause death; (B) Cardiovascular death.

The Kaplan-Meier curves for survival by quartiles of combined Cr and TBil values are shown in Fig 2, with all-cause death as the outcome event, and the median survival times for the four groups were 3.95 (95% CI: 3.29–4.93) years, 3.21 (95% CI: 2.96–3.70) years, 3.21 (95% CI: 2.88–3.70) years, and 2.00 (95% CI:1.81–2.16) years (log-rank test, P<0.0001). With cardiovascular death as the outcome event, the median survival time was 4.05 (95% CI:3.37–5.08) years, 3.21 (95% CI:2.96–3.78) years, 3.35 (95% CI:2.96–3.86) years, and 2.05 (95% CI:1.83–2.22) years in the four groups, respectively (log-rank test, P<0.0001). The survival rate of group 4 IHD patients was significantly lower than the rest of the groups, both in all-cause and cardiovascular death events **(Fig 2)**.

### Association of Cr, TBil, and combined value with long-term death

**Fig 3:**
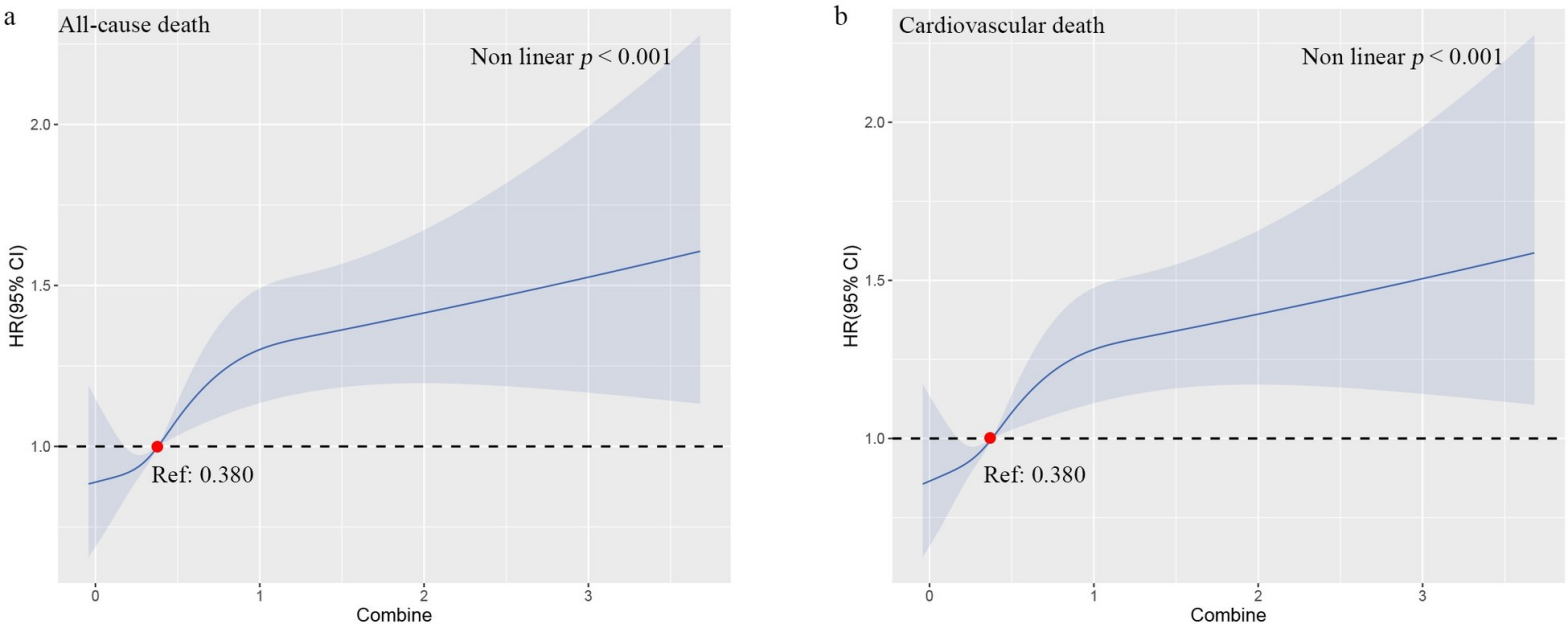
Restricted cubic spline (RCS) plot of the association between the combined value of Cr and TBil with the risk of long-term death in IHD patients. (A) All-cause death; (B) Cardiovascular death. Blue lines represent references for HRs, and blue areas represent 95% confidence intervals. The model was adjusted for gender, age, BMI, smoking, drinking, hypertension, diabetes, albumin, left ventricular ejection fraction, fasting plasma glucose, triglyceride, total cholesterol, admission of myocardial infarction, heart failure, arrhythmia, and past percutaneous coronary intervention.

**Fig 4:**
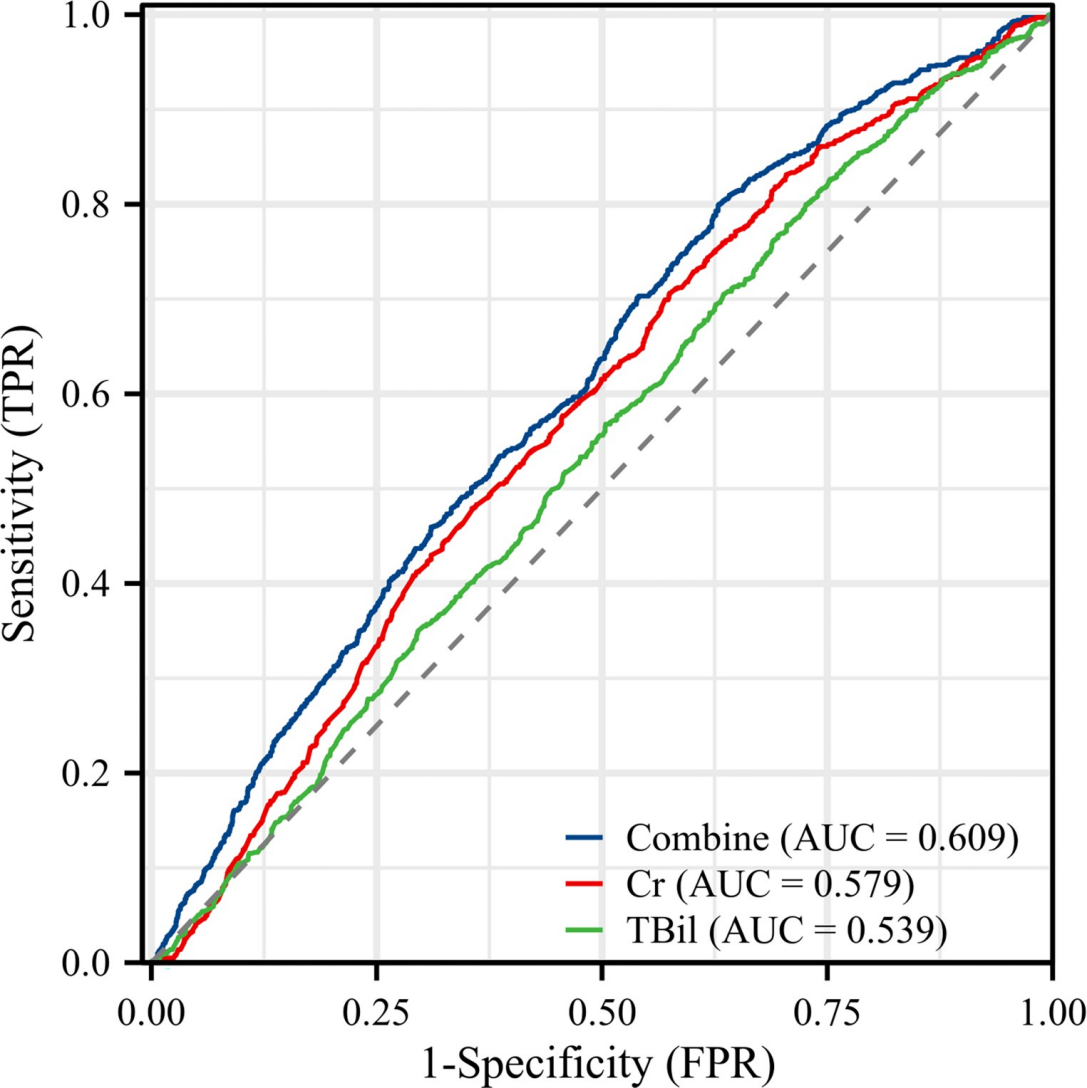
Receiver operating characteristic (ROC) curves of Cr, TBil, and combined value as markers to predict all-cause death in patients with IHD.

To observe the relationship between Cr, TBil, and combined value with long-term death events, we performed univariate and multifactor Cox proportional risk regression analyses with all-cause death and cardiovascular death as dependent variables, respectively. With all-cause death as the outcome event, Cr (1.002 (1.002–1.003), P<0.001) and TBil (1.007 (1.004–1.010), P<0.001) were both found to be risk factors for all-cause death in model 1 (i.e., univariate Cox regression analysis). Models 2, 3, and 4 showed that the hazard ratios (HRs) for Cr (Model 4: 1.001(95% CI: 1.000–1.002), P<0.05) and TBil (Model 4: 1.007(95% CI: 1.004–1.010), P<0.05) remained statistically significant after adjusting for numerous confounding factors. In the four groups divided by quartiles of combined value, the results of the multi-model Cox regression analysis with group 1 as reference showed that the unadjusted HRs for all-cause death were 1.199 (95% CI: 1.036–1.388), 1.224 (95% CI: 1.058–1.416), 2.015 (95% CI. 1.754–2.315), and in model 4, which adjusted for the most confounding factors, the adjusted HRs for all-cause death in groups 2, 3 and 4 were 1.145 (0.987–1.330), 1.082 (0.930–1.258) and 1.457 (1.256–1.689), respectively, and the increased risk of all-cause death from group 2 to group 4 in each model was statistically significant (P for trend<0.001). Similar results were obtained from Cox proportional risk regression analysis with cardiovascular death as the outcome event **(Table 3)**. In addition, the RCS regression models showed a nonlinear relationship with the combined value between all-cause death and cardiovascular death, with an increased risk of death occurring with higher combined values (nonlinear P < 0.001) **(Fig 3)**. The results of ROC curve analysis showed that the AUCs of Cr, TBil, and combined value for predicting long-term all-cause death events in patients with IHD were 0.579 (95% CI: 0.557–0.601, P<0.001), 0.539 (95% CI: 0.517–0.562, P<0.001), and 0.609 (95% CI: 0.587–0.630, P<0.001), with sensitivities of 70.6%, 80.0%, and 80.0%, and specificities of 42.5%, 27.4%, and 37.1%, respectively **(Fig 4 and Table 4)**.

**Table 3:** Cox proportional hazard regression analyses of Cr, TBil and combined value for long-term death.

Variable	HR (95% CI)			
	Model 1	Model 2	Model 3	Model 4
All-cause death				
Cr (per 1 unit increase)	1.002(1.002–1.003) ***	1.002(1.001–1.002) ***	1.001(1.001–1.002) ***	1.001(1.000–1.002) *
TBil (per 1 unit increase)	1.007(1.004–1.010) ***	1.009(1.006–1.011) ***	1.007(1.004–1.010) ***	1.007(1.004–1.010) *
Combine				
Group 1	ref.	ref.	ref.	ref.
Group 2	1.199(1.036–1.388) *	1.201(1.036–1.393) *	1.154(0.994–1.340)	1.145(0.987–1.330)
Group 3	1.224(1.058–1.416) *	1.209(1.042–1.403) *	1.100(0.946–1.279)	1.082(0.930–1.258)
Group 4	2.015(1.754–2.315) ***	1.723(1.493–1.989) ***	1.494(1.289–1.731) ***	1.457(1.256–1.689) ***
P for trend	<0.001	<0.001	<0.001	<0.001
Cardiovascular death				
Cr (per 1 unit increase)	1.003(1.002–1.003) ***	1.001(1.001–1.002) ***	1.001(1.001–1.002) ***	1.001(1.000–1.002) *
TBil (per 1 unit increase)	1.007(1.004–1.010) ***	1.009(1.007–1.012) ***	1.008(1.005–1.011) ***	1.007(1.004–1.011) ***
Combine				
Group 1	ref.	ref.	ref.	ref.
Group 2	1.221(1.048–1.424) *	1.219(1.043–1.424) *	1.169(0.999–1.366)	1.157(0.989–1.353)
Group 3	1.230(1.055–1.434) *	1.215(1.040–1.421) *	1.106(0.944–1.296)	1.085(0.926–1.271)
Group 4	2.036(1.760–2.355) ***	1.731(1.490–2.010) ***	1.495(1.282–1.744) ***	1.452(1.244–1.695) ***
P for trend	<0.001	<0.001	<0.001	<0.001

Abbreviations: HR, hazard ratio; 95% CI, 95% confidence interval; Cr, creatinine; TBil, total bilirubin.

Patients were divided into four groups according to the quartiles of combined value: Group 1: Combine ≤ 0.236; Group 2: 0.236 < Combine ≤ 0.381; Group 3: 0.381< Combine ≤ 0.592; Group 4: Combine > 0.592.

Model 1: Unadjusted.

Model 2: Adjusted for gender, age, BMI, smoking, drinking, hypertension, and diabetes.

Model 3: Adjusted for gender, age, BMI, smoking, drinking, hypertension, diabetes, albumin, LVEF, FPG, TG, and TC.

Model 4: Adjusted for gender, age, BMI, smoking, drinking, hypertension, diabetes, albumin, LVEF, FPG, TG, TC, MI, HF, arrhythmia, and past PCI.

Significance markers

*p<0.05

***p<0.001

**Table 4:** AUCs of Cr, TBil and combined value as predictors of all-cause death.

Variable	AUC (95%CI)	Sensitivity	Specificity	Youden index	Cut-Off Value
Cr	0.579(0.557–0.601) ***	0.706	0.425	0.131	94.920
TBil	0.539(0.517–0.562) ***	0.800	0.274	0.074	20.120
Combine	0.609(0.587–0.630) ***	0.800	0.371	0.170	0.527

Abbreviations: AUC, area under the curve; Cr, creatinine; TBil, total bilirubin.

Significance markers

***p<0.001

## Discussion

This study shows for the first time that combined value of Cr and TBil exhibit a stronger risk relationship with long-term death events in patients with IHD compared with single factors, and that the combination of the two better predicts the occurrence of long-term death in patients with IHD. Our study showed that patients with higher levels of both Cr and TBil had higher long-term death, although after adjusting for numerous confounding factors.

The pathophysiologic mechanism of IHD is more complex than a single, simple causal event. It can develop from coronary microvascular dysfunction, inflammation, or vasospasm, but more often it is generally an obstruction or stenosis of the coronary arteries due to atherosclerotic lesions that obstruct coronary circulation flow and inadequate myocardial blood supply, which in turn develops into IHD. It has been shown that in the general population, elevated plasma Cr levels are associated with an increased risk of early death from ischemic heart disease, whereas low levels of glomerular filtration rate were not significantly correlated [5, 10]. Mechanistic studies suggest that creatinine may contribute to the progression of ischemic heart disease through several pathways, including increasing the extent and number of atherosclerotic lesions, increasing the number of low-density lipoprotein particles, and vascular inflammation [11, 12]. Elevated Cr levels may alter protein turnover, and proteinuria leads to the retention of residual cholesterol from the degradation of chylomicron and very low-density lipoprotein cholesterol particles in plasma [13], and the accumulation of residual cholesterol can inhibit interleukin-10 production. Produced mainly by activated helper T cell subsets Th2 cells, B cells, and monocytes/macrophages, interleukin-10 inhibits the prototypical pro-inflammatory transcriptional nuclear factor-kB, which in turn inhibits inflammatory cytokine production, inhibits matrix-hydrolyzed metalloproteinases, attenuates tissue factor expression, promotes a shift in lymphocyte phenotype toward Th2 cells, and overall suppresses the inflammatory response [14, 15]. IL-10 also has a potential anti-atherosclerotic effect, as IL-10 inactivates local macrophages and T lymphocytes and can modulate the local inflammatory response [16, 17].

High levels of Cr are often accompanied by decreased renal function in patients, and chronic kidney disease is a common co-morbidity in patients with IHD and is associated with worse short- and long-term clinical prognosis [18–20], and patients with chronic kidney disease exhibit accelerated atherosclerosis and an increased risk of multivessel coronary artery disease [21], which may explain the increased risk of death in IHD patients with high levels of Cr. Susanne et al. found in animal experiments that chronic renal failure accelerated the process of atherosclerosis and that mice with chronic renal failure had higher plasma total cholesterol concentrations than control mice, a difference that likely contributed to their accelerated atherosclerosis formation [11]. Julio et al. performed a mortality risk analysis of 122 patients with ischemic cardiomyopathy and similarly obtained a trend of higher mortality associated with higher Cr levels [22]. Joachim et al. showed that in patients with coronary artery disease, Cr excretion rates were strongly associated with their mortality, and they found that lower Cr excretion rates corresponded to a higher risk of death (HR:2.30 (95% CI: 1.51–3.51), P = 0.001), after adjusting for various confounders [23].

In addition to the pathophysiological mechanisms analyzed above, oxidative stress and inflammatory responses play a large role in the development of the IHD disease process. Hemoglobin is highly reactive in its unbound form. In the free state, highly reactive heme accelerates the production of reactive oxygen species and lipid oxidation, thus increasing the risk of cardiovascular disease [24, 25]. Heme oxygenase (HO) cleaves the pro-oxidant heme on the α-methylene bridge to form biliverdin, carbon monoxide, and ferrous iron. The biliverdin is then reduced to bilirubin by biliverdin reductase. Thus, HO has antioxidant and anti-inflammatory effects [26, 27]. A study by Ozturk et al. of 782 patients with acute coronary syndrome found a significant positive correlation between TBil levels and troponin levels at admission. They hypothesized that HO-1 is a stress-inducing enzyme and that the increase in HO-1 activity during acute myocardial infarction corresponds to elevated TBil levels [28]. Okuhara et al. found a significant positive correlation between HO-1 enzyme levels and TBil levels at admission in patients with acute myocardial infarction, further supporting the argument of Ozturk et al. and also suggesting that elevated serum TBil reflects HO-1 activation [7]. Elevated levels of the HO-1 enzyme represent the presence of high-intensity oxidative stress and inflammatory response in the body, and high levels of HO-1 correspond to high levels of TBil. Therefore, we hypothesize that the degree of HO-1 activation may reflect the intensity of the inflammatory response to myocardial injury during the course of IHD and that high levels of TBil suggest an increased risk of adverse cardiovascular events.

Halit et al. reported that patients with acute myocardial infarction with impaired blood flow had higher bilirubin levels than the group with normal blood flow, suggesting that the more severe the degree of atherosclerosis and the higher the post-infarction HO-1 enzyme activity, the more marked the increase in bilirubin levels, and the degree of increase was related to the severity of the lesion [29]. Huang et al. showed that serum TBil levels were associated with higher acute myocardial infarction group mortality (OR: 2.35, 95% CI: 1.15–4.77, P<0.05) [30]. Chung et al. followed up on 1,111 patients with ST-segment elevation infarction in-hospital and 12 months postoperatively. The results showed that the incidence of adverse cardiovascular events and cardiac mortality were higher in the hyperbilirubinemic group than in the hypobilirubinemic group [31]. This suggests that oxygen radicals and various oxidants produced by oxidative stress injury may damage the organism under stressful conditions more than the anti-inflammatory and antioxidant effects of HO-1 on the organism, and high levels of TBil instead show a strong correlation with poor prognosis.

In summary, not only one system or one pathophysiological mechanism is involved in the disease process of IHD, and a single inflammatory biomarker or oxidative stress alone is not sufficient to elucidate the entire pathophysiological process involved in IHD. On the contrary, combining multiple markers can provide a more comprehensive picture of the developmental process of IHD.

In the present study, the area under the ROC curve values for the combined equations predicting mortality, although not very high, was still higher than the AUC values predicted by Cr and TBil alone, respectively. More importantly, multifactorial COX regression analysis showed that high values of the equation were independent predictors of out-of-hospital long-term death events. Despite adjusting for numerous confounders, the combined value of Cr and TBil exhibited a high mortality risk correlation compared with the separate metrics. This suggests that combining Cr and TBil is a stronger predictor of long-term death events than the markers alone. And since Cr and TBil, two rapidly available biomarkers commonly used in routine blood tests, are suitable for most hospitals due to their low cost and ease of detection, we still suggest that the combined use of Cr and TBil is of particular clinical importance for predicting long-term prognosis in patients with IHD.

### Advantages and limitations

The strength of this study is the use of two biomarkers that are readily available in the clinical setting combined with regression equations to obtain a highly correlated and more sensitive indicator of the risk of death from IHD. However, the present study still has some limitations. This study is a single-center trial with the limitations of a retrospective cohort design. Plasma Cr and TBil levels vary over time, change with diet, and are affected by medications; therefore, a single measurement provides an insensitive indicator and should be compared to repeated measurements. This study did not directly measure oxidative stress-related indicators and HO-1 enzyme activity, nor did it include various inflammatory markers such as C-reactive protein, calcitonin gene, and interleukins. More basic experimental studies are needed to validate the exact role of Cr and TBil in the long-term prognosis of patients, in addition to determining the exact role of Cr and TBil.

## Conclusion

This study showed that the combined Cr and TBil assay is superior to single biomarkers for predicting out-of-hospital long-term death events in patients with IHD. High values of the equation are independent predictors of out-of-hospital long-term death events and can be used to identify patients at high risk for IHD and accurately predict their clinical prognosis for early intervention.

## Supporting information

S1 Data The dataset used in this paper. (XLSX)
